# A Novel Privacy Preservation and Quantification Methodology for Implementing Home-Care-Oriented Movement Analysis Systems

**DOI:** 10.3390/s22134677

**Published:** 2022-06-21

**Authors:** Pablo Aqueveque, Britam Gómez, Patricia A. H. Williams, Zheng Li

**Affiliations:** 1 Electrical Engineering Department, Universidad de Concepción, Concepción 4070409, Chile; pablo.aqueveque@biomedica.udec.cl (P.A.); britam.gomez@biomedica.udec.cl (B.G.); 2 College of Science and Engineering, Flinders University, Adelaide, SA 5042, Australia; trish.williams@flinders.edu.au; 3 Department of Computer Science, Universidad de Concepción, Concepción 4070409, Chile

**Keywords:** movement analysis, inertial movement units, risk of falls, gait analysis, privacy preservation, privacy quantification, privacy risks, patient-centric healthcare

## Abstract

Human movement is generally evaluated through both observations and clinical assessment scales to identify the state and deterioration of a patient’s motor control. Lately, technological systems for human motion analysis have been used in clinics to identify abnormal movement states, while they generally suffer from privacy challenges and concerns especially at home or in remote places. This paper presents a novel privacy preservation and quantification methodology that imitates the forgetting process of human memory to protect privacy in patient-centric healthcare. The privacy preservation principle of this methodology is to change the traditional data analytic routines into a distributed and disposable form (i.e., DnD) so as to naturally minimise the disclosure of patients’ health data. To help judge the efficacy of DnD-based privacy preservation, the researchers further developed a risk-driven privacy quantification framework to supplement the existing privacy quantification techniques. To facilitate validating the methodology, this research also involves a home-care-oriented movement analysis system that comprises a single inertial measurement sensor and a mobile application. The system can acquire personal information, raw data of movements and indexes to evaluate the risk of falls and gait at homes. Moreover, the researchers conducted a technological appreciation survey of 16 health professionals to help understand the perception of this research. The survey obtains positive feedback regarding the movement analysis system and the proposed methodology as suitable for home-care scenarios.

## 1. Introduction

The healthcare industry’s digital transformation can be traced back to the development of computer engineering and information and communication technologies (ICT) in the 1950s [1]. Driven by the revolutionary development of modern computing devices, i.e., from large mainframes to small chips that can be embedded in wearable objects and sensors, healthcare is experiencing a rapid evolution in the use of Internet of Things (IoT) [2]. As such, it has been predicted that healthcare provisioning will transform from the current hospital-centred approach, first to the hospital-home-balanced hybrid model in the 2020s, and subsequently to a primarily home-centred model in the 2030s [3,4,5]. Compared to the traditional healthcare delivery model and hospital-oriented systems, the emerging personalised paradigm has been identified as able to improve preventive healthcare by increasing people’s awareness of, and involvement in, the management and maintenance of their health [6].

A typical use case of the home-centred healthcare model is the individuals’ movement analyses. In clinical movement analysis, health professionals not only need observations but also test different bio-mechanical aspects to assess the state and deterioration of a patient’s motor control over various activities [7,8]. In recent years, high-technological systems have been employed for the bio-mechanical evaluation of movement, such as high-resolution camera-based systems, pressure mats, electromyography systems and balance platforms. Those systems can help characterise or predict the gait quality, risk of falls, comorbidities, balance and the patient dependence [9,10,11,12,13,14].

When implementing movement analysis systems in the home environments, there are inevitable privacy challenges and concerns. In fact, it has been identified that privacy is specifically critical in the home-based and/or wearable systems [6,15]. Several exploratory studies indicate that the main concerns about the privacy when using wearable systems include: the lack of control over the collected and shared information, unexpected disclosure of the user identification, and the misuse of the collected data [16,17]. Thus, many wearable systems have incorporated hardware/software solutions to protect privacy, such as using audio-visual feedback [18], physical and gesture switches to stop recording [19], using identification masking when information is being shared [20], and using machine learning algorithms to remove sensitive information automatically [21]. However, most of these solutions allow only to control the information to be masked or to decide when it is sent or recorded, while they do not allow users to repeatedly choose what, how, with whom, and when to store, process and transmit data in a consistent methodology.

Driven by such a gap, we have developed a novel methodology that enabled a distributed and disposable approach (DnD) to exclude patients’ sensitive information from health data analytics. Recall that a methodology refers to “an organised set of principles which guide action in trying to ‘manage’ (in the broad sense) real-world problem situations” [22]. In the DnD-applicable cases, we advocate for *forgetting while analysing* as an intrinsic privacy preserving principle in patient-centric healthcare. As such, raw data with sensitive information will be immediately “forgotten” (neither stored nor transmitted) after their usage in the owner-side analytic, which imitates the natural forgetting process of human memory. Based on our proof-of-concept development and application of DnD within the home-care scenarios [23], this research further reinforces the theoretical foundation of DnD. Firstly, we have enriched DnD-friendly data analytic methods and followed the open method principle to share the method details [24]. Secondly, to help judge the efficacy of DnD (and other privacy-preserving techniques), we have developed an innovative privacy quantification framework based on fuzzy logic and privacy risk analysis. By quantitatively distinguishing between the privacy levels/risks within different data analytical scenarios, this methodology can conveniently and appropriately integrate the existing privacy preservation techniques, even if they focus on the minor and unusual aspects of privacy rather than addressing the essential aspects [25].

To verify the practical effectiveness and efficiency of our methodology, we also developed a home-care-oriented movement analysis system based on a wearable magneto-inertial measurement unit (MIMU). Technically, the MIMU sends raw data (tri-axial acceleration, tri-axial angular velocity, and tri-dimensional orientations) to a mobile platform to calculate clinical assessment scales for analysing the risk of falls and the gait quality [26,27]. The advances in movement analysis algorithms and device miniaturization make this system a low-cost and easy-to-use alternative to those high-technological systems of specialised laboratories for the bio-mechanical analysis of movement [28]. By introducing the movement analysis system together with our privacy preserving methodology to several local patient-centric healthcare projects, we have received positive feedback from the domain professionals.

Based on our local validation, we claim that this research particularly contributes to the remote healthcare for elderly people. According to the WHO, population ageing is happening and accelerating unprecedentedly [29]. The estimation is that the global number of people aged 60 years and older will increase to 1.4 billion by 2030 and 2.1 billion by 2050 [29]. It is known that human ageing inevitably increases the risk of disability, dependence, and a number of comorbidities. In addition, many seniors could stay lonely and isolated especially in developing countries, which further increases the aforementioned risks. Therefore, it will be significantly valuable and helpful to offer home-care-oriented monitoring services to those elderly individuals, while this research can conveniently be applied and/or adapted to those multi-purpose daily activity monitoring systems.

The remainder of this paper is organised as follows. Section 2 briefly explains DnD-based privacy preservation and mainly introduces the newly developed privacy quantification framework. To facilitate applying DnD, a set of DnD-friendly data analytic methods are detailed in Section 3. Section 4 describes a home-care-oriented movement analysis system for validating our privacy preservation and quantification methodology. Section 5 demonstrates how we can apply DnD and the privacy quantification framework to the home-care scenarios, especially for caring elderly people. Section 6 draws conclusions and discusses our future work.

## 2. Privacy Preservation and Quantification for Implementing Movement Analysis Systems for Home Care

As mentioned earlier, the home-based healthcare provisioning inevitably involves more privacy risks than the hospital mode does. To address the privacy concerns when implementing movement analysis systems for home care, we introduce a novel privacy-preserving methodology that includes a DnD approach to privacy preservation and a privacy quantification framework.

### 2.1. DnD-Based Privacy Preservation

Paralleling the digital transformation of healthcare, privacy issues inevitably arise as a major concern in the healthcare ecosystem [25]. These issues are apparent, specifically in the IoT-based patient-centric context [6] and in the big data era [15]. Patients’ privacy can be breached generally by the leakage of their health data, as the leakage may expose sensitive medical histories and other private information [30]. As a result, it has been reported that health data leakage can not only incur huge monetary losses but also cause significant negative impacts on individuals and the society [31]. Despite the decades of effort in digital healthcare system development, privacy studies investigating the exploitation of big healthcare data are still at an early stage. People expect government regulations and trusted organisations to mitigate privacy problems [6], yet the current legal frameworks have lagged behind the development of technologies and the business of big data analytics [32]. Although there exist sophisticated techniques of privacy preservation, these are generally inadequate for the emerging big data era [25], focusing on minor and unusual aspects of privacy rather than addressing the essential aspects.

Driven by the privacy concerns together with the IoT characteristics (i.e., the generally limited compute, storage and network resources of IoT devices), we have proposed a DnD approach to privacy preservation [23]. The DnD approach is developed based on the following intuitive and straightforward consensus:

- The less data/information we share, the less privacy risks we take.
- The less data/information we store, the less privacy risks we take.

It should be noted that DnD is not intended to replace the existing privacy-preserving mechanisms but to provide an additional layer to suitable techniques to further enhance the privacy-preserving effects. For example, the differential privacy preservation mechanism [33] matches one of the DnD scenarios where the raw data are excluded (or removed) from the analytical results. The discussion about, and the development of, DnD have been elaborated in our earlier work [23]. To avoid duplication, we do not repeat them in this paper. Instead, we directly show the application of DnD in Section 5.1, while this paper mainly describes our newly developed privacy quantification framework in the following subsection.

### 2.2. Risk-Driven Privacy Quantification Framework

**Figure 1:**
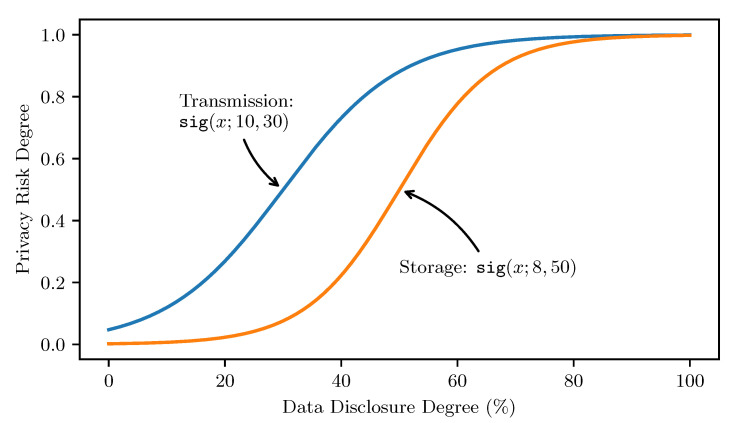
Two potential fuzzy membership functions for estimating and measuring privacy risks in different data contexts. (See their explanation and usage in Section 5.3.)

The development of the DnD corresponds to a common concern around the linguistic variable “privacy risk”; i.e., the more health data people disclose (store or transmit to the outside world), the higher privacy risks people take. It is clear that estimating privacy risk degrees is more straightforward than directly estimating privacy levels in human discussions. Therefore, this research uses privacy risks as a bridge to investigate privacy quantification. Considering the subjective and imprecise descriptions on privacy risk degrees in natural language, this research uses fuzzy logic to grade the fuzzy set of linguistic terms of privacy risk (e.g., from “extremely low” to “extremely high”). Specifically, given the generally positive proportional relationship between data disclosure and privacy risk, the Sigmoidal (“s-shaped”) Membership function [34] is used to represent the gradual transition between those linguistic terms, as shown in Equation (1).

(1) $$\mathrm{sig}\left(x;s,p\right)=\frac{1}{1+e^{-(x-p)/s}},\;1\leq s\leq 10,\;0<x,p<100$$

where the membership function sig(x;s,p) delivers the privacy risk degree between 0.0 and 1.0 when *x* percent of data are disclosed; *p* indicates the environment’s (or data context’s) objective privacy sensitivity, which can be adjusted as the inflection point when reaching the privacy risk degree 0.5; and *s* represents the user’s subjective privacy sensitivity from 1 to 10, which can be obtained through questionnaires case by case. Within a particular environment, the higher the privacy sensitivity, the faster the privacy risk increases along with the data disclosure. For a single person, higher privacy sensitivity implies less tolerance in disclosing personal data and thus brings higher privacy risks to him/her even when disclosing a small amount of data. By assuming the privacy sensitivities are 10 and 8 in two different data contexts, respectively, and the inflection points are correspondingly at 30% and 50% of data disclosure, we demonstrate two concrete fuzzy membership functions for estimating and measuring privacy risks, as illustrated in Figure 1.

Given a specific degree of privacy risk, we implement privacy quantification by taking advantage of the negative proportional relationship between privacy risk and privacy level, as defined in Equation (2).

(2) P=1−sig(x;s,p)

where P represents the quantified privacy, and it can also be explained as the confidence in privacy preservation within a particular situation. Such a definition fits in the common sense: fewer privacy risks usually imply a better privacy environment with higher privacy levels.

Since different privacy risks exist in different data contexts (e.g., data storage and data transmission), the risk analysis and privacy quantification need to be conducted independently in each context. By treating independent data contexts as orthogonal privacy dimensions, we formulate a generic privacy quantification framework into Equation (3).

(3) $$P=\prod_{k=1}^{m}P_{k}$$

where P represents the combined confidence in privacy preservation across *m* data contexts with respect to their individual privacy levels $P_{1},P_{2}\ldots ,$ and $P_{m}$. As mentioned previously, the current version of DnD takes into account privacy only in data storage and data transmission. As such, the privacy quantification under DnD can be generalised as $P_{DnD}=P_{storage}\times P_{transmission}$. The detailed implementations are exemplified together with a case study in Section 5.3.

### 2.3. Comparison against the Existing Privacy Quantification Approaches

In addition to qualitative discussions, privacy quantification is often needed to measure the privacy levels in different environments or situations (e.g., before and after applying privacy-enhancing technologies) in order to compare and judge the effectiveness and efficiency of different privacy-preserving techniques [35]. In other words, privacy quantification also plays a crucial role in privacy preservation. Here, we briefly and critically review four de facto approaches to privacy quantification to help justify the development of our privacy quantification framework.

- The **Information–Theoretic Approach** takes advantage of the inherent connection between privacy and information from a mathematical perspective, and it quantifies privacy by measuring the amount of information lost by users or the amount of information gained by attackers after data disclosure. Intuitively, the smaller the attacker’s gain or the user’s loss of information, the higher the privacy level. Based on the matured information theory, this approach has widely been discussed in academia. However, the sophisticated mathematical basis of this approach makes it hard to adapt to the diverse industrial needs [36], and it demonstrates the large gap between mathematical and natural languages when conducting empirical studies. For example, the concept “entropy” seems particularly difficult for normal people (e.g., patients) to understand and use in practical communications.
- The **Uncertainty-Based Approach** integrates an uncertainty data model into a privacy model, so as to quantify privacy with regard to the degree of uncertainty at which the private data can be inferred [37]. The quantification logic is that the higher the degree of uncertainty (e.g., achieved by a privacy preserving technique), the better the privacy in the protected data. Nevertheless, it has been identified that uncertain information-based guesses can still be correct, and consequently, privacy breaches may still occur even under highly uncertain circumstances [35].
- The **Input–Output Similarity-based Approach** utilises the similarity or diversity between the original data and the processed (or queried) data to quantify privacy, and it is primarily related to the anonymisation techniques in the database domain [38]. In this way, high privacy can usually be reflected by low similarity or high diversity between the input and output of data processing. This approach focuses on the data changes without considering the data disclosure environment; however, this will overestimate the privacy levels in some cases. For example, regular patterns in the anonymised data would still affect privacy if attackers were equipped with relevant background knowledge.
- The **Output–Output Indistinguishability-Based Approach** quantifies privacy by measuring the degree of obfuscation between the output data from different (while linked or relevant) data processing events [39]. Accordingly, the harder it is to identify the relevance between related processing outcomes, the higher the privacy. It is clear that the privacy quantification here requires at least a pair of output datasets, and thus, this approach is not suitable to help understand the privacy levels within standalone patient-centric events.

To address the major concerns in the existing privacy quantification approaches, this research focuses on the linguistically easy-to-understand “privacy risk” and investigates fuzzy reasoning to encode human knowledge or common sense into numerical privacy recognitions, as demonstrated in Section 5.3. In particular, compared with the information–theoretic approach, the fuzzy-logic basis can make privacy risk-driven solutions easily adaptable to various data contexts via natural-language discussions. Compared with the input–output similarity-based approach, this research takes into account not only the data disclosure but also the influences from the environment and the patient.

## 3. DnD-Friendly Data Analytic Methods

We have identified that running statistics and streaming algorithms [40] are particularly suitable for DnD analytics. Unlike traditional methods that require preparing full data sets and correspondingly require large memory/storage spaces, both running analytics and streaming algorithms employ a recursive approach to the analytic computation without necessarily storing any historical data. Thus, these methods can be more efficient and lightweight than their traditional counterparts, especially in the real-time analytic situations. More importantly, the recursion feature matches the disposable fashion advocated by DnD for privacy preservation by avoiding storing and summing up all the data in the brute-force way.

The current stage of the research primarily uses “running analytical” methods for the development of DnD. The performance benefits of using running analytics have been noted over several decades [41]. Nevertheless, the implementations of running analytics are minimal [42,43] except for a couple of simple cases (i.e., running average and running variance). Even in the simple case of the running variance, there exist inconsistent implementations that either violate the spirit of recursion or lack arithmetic proofs. For example, the implementation in [44] is based on data accumulation, while “it is not obvious that the method is correct even in exact arithmetic” in [45].

To address the issues in the use of running analytics, we have investigated and shared the details of the typical running analytic methods employed in this research, including running min, max, average, sample standard deviation, and skewness. In fact, making analytic methods openly available has been identified as a higher-level strategy over open-source tools and open-access data for improving the repeatability, replicability, and reproducibility of scientific work [24]. It is noteworthy that some other analytic methods (e.g., Coefficient of Variance) can directly be implemented based on these typical ones. Furthermore, we have taken into account not only the arithmetic correctness but also the computation efficiency (e.g., avoiding the repeated calculations of intermediate results) through this research. As justified in [24], the shared formulas together with their detailed proofs (the detailed proofs of the methods specified in Section 3.1 are published in a separate paper [46]) can significantly facilitate the improvement and/or modification of those methods, for example, when uneven time intervals need to be considered in data analytics.

### 3.1. An Extendable Suite of Analytic Methods for Implementing DnD

According to our recent projects, the following statistical methods are most commonly used in the home-care scenarios.

#### 3.1.1. Running Min and Running Max

Both minimum and maximum thresholds of many health indicators (e.g., blood pressure and heart beat rate) are useful to generate early warnings in patient-centric healthcare systems. For example, the value of oxygen saturation (SpO2) below 90% has been used for asymptomatic patients to detect COVID-19 infection [47]. Thus, running min and running max are defined as two simple while crucial analytic methods in this work, as shown in Equations (4) and (5).

(4) $$min_{n+1}=\min\left(min_{n},x_{n+1}\right),\;min_{0}=0$$

(5) $$max_{n+1}=\max\left(max_{n},x_{n+1}\right),\;max_{0}=0$$

where $min_{n}$ and $max_{n}$, respectively, indicate the minimum and maximum values within a series of data $x_{1},x_{2},\ldots ,x_{n}$. After the new value $x_{n+1}$ joins the data series, the functions min() and max() will make comparisons and return the new minimum and maximum values $min_{n+1}$ and $max_{n+1}$.

#### 3.1.2. Running Average

In medical examination reports, average measurement results with respect to various indicators are widely used to reflect a patient’s health status. When conducting DnD analytics, the running average can be calculated by Equation (6).

(6) $$\mu_{n+1}=\mu_{n}+\frac{x_{n+1}-\mu_{n}}{n+1},\;\mu_{0}=0,\;n>0$$

where $\mu_{n+1}$ indicates the arithmetic average of n+1 values after the new value $x_{n+1}$ joins the data series.

#### 3.1.3. Running Sample Standard Deviation

The measure of standard deviation is commonly used to quantify how far a set of data spreads out from its average value. To facilitate the corresponding programming, this research highlights the recursive calculation of sample variance, as shown in Equation (7).

(7) $$\left\{ \begin{array}{c} \begin{array}{c} V_{n+1}=V_{n}+\left(x_{n+1}-\mu_{n+1}\right)\left(x_{n+1}-\mu_{n}\right) \end{array}\\ \sigma_{n+1}^{2}=\frac{V_{n+1}}{n},\;V_{0}=V_{1}=0,\;n>1 \end{array}\right.$$

where $\sigma_{n+1}^{2}$ represents the unbiased estimator for the sample variance, after the new value $x_{n+1}$ joins the data series. $\sigma_{n+1}$ is then the corresponding standard deviation. In particular, the sample variance $\sigma_{n}^{2}$ is defined as a proportional function of the auxiliary variable $V_{n}$ that can be calculated in a recursive way, while $V_{n}$ essentially carries the crucial information of the second Central Moment, i.e., $\sum_{i=1}^{n}{\left(x_{i}-\mu_{n}\right)}^{2}$.

#### 3.1.4. Running Sample Skewness

When focusing on the value space of a series of random data, skewness measures or estimates the symmetry of the data’s distribution by comparing to the corresponding normal distribution. The recursive calculation formula of sample skewness is given by Equation (8).

(8) $$\left\{ \begin{array}{c} S_{n+1}=S_{n}-3\cdot \left(\mu_{n+1}-\mu_{n}\right)\cdot V_{n}+\left(V_{n+1}-V_{n}\right)\left[x_{n+1}-\mu_{n+1}-\left(\mu_{n+1}-\mu_{n}\right)\right]\\ S_{n+1}=\frac{\left(n+1\right)\cdot S_{n+1}}{n\cdot \left(n-1\right)\cdot \sigma_{n+1}^{3}},\;\;\;\;\;\;S_{0}=S_{1}=S_{2}=0,\;n>2 \end{array}\right.$$

where $S_{n+1}$ indicates the sample skewness of n+1 values, after the new value $x_{n+1}$ joins the data series. In particular, $S_{n}$ is defined as a proportional function of the auxiliary variable $S_{n}$ that can be calculated in a recursive fashion, while $S_{n}$ essentially carries the crucial information of the third Central Moment, i.e., $\sum_{i=1}^{n}{\left(x_{i}-\mu_{n}\right)}^{3}$.

It should also be noted that we have intentionally prepared these equation forms for improving algorithm efficiency. When implementing these analytic methods, practitioners can employ temporary variables to reuse suitable intermediate results to minimise computational workloads and to speed up programs. For example, $\left(\mu_{n+1}-\mu_{n}\right)$ and $\left(V_{n+1}-V_{n}\right)$ in Equation (9) can directly be obtained from the intermediate results in Equations (6) and (7).

## 4. A Wearable Movement Analysis System for Home Care

To facilitate validating our privacy preservation and quantification methodology, we further developed a wearable movement analysis system. The developed system consists of a wireless sensor for movement analysis and a mobile application that controls, receives and processes the raw data from the wireless sensor, incorporating clinical tests for the evaluation of fall risk and gait quality.

### 4.1. Electronic Sensor to Monitor Human Movement

We design an magneto-inertial measurement unit (MIMU) using a BNO055 sensor from Bosh Sensortec. This MIMU has a three-axis accelerometer, a three-axis gyroscope, and a three-axis magnetometer, as well as an internal processor capable of merging the inertial and magnetic data using an extended Kalman filter to deliver the orientation in quaternions.

The inertial and magnetic variables are measured at a sample frequency of 100 Hz using a STM32 32-bit microcontroller with an ARM Cortex-M4 processor. The raw data are sent to a mobile app over a Bluetooth 5.0 link. We use a 3.7 V, 1200 mAh LiPo battery to power the entire system, which gives the sensor up to 30 hours for continuous measurements.

**Figure 2:**
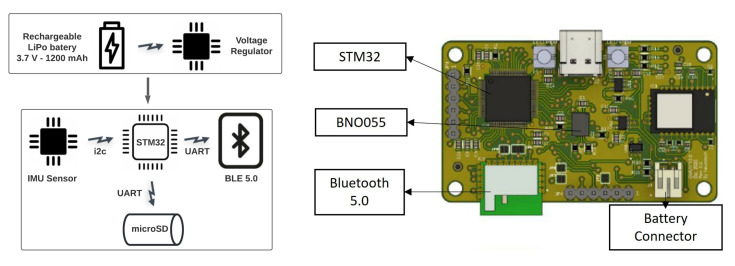
General electronic scheme and electronic PCB design of the implemented motion sensor.

A simplified block diagram and electronic PCB design of the sensor are shown in Figure 2.

**Figure 3:**
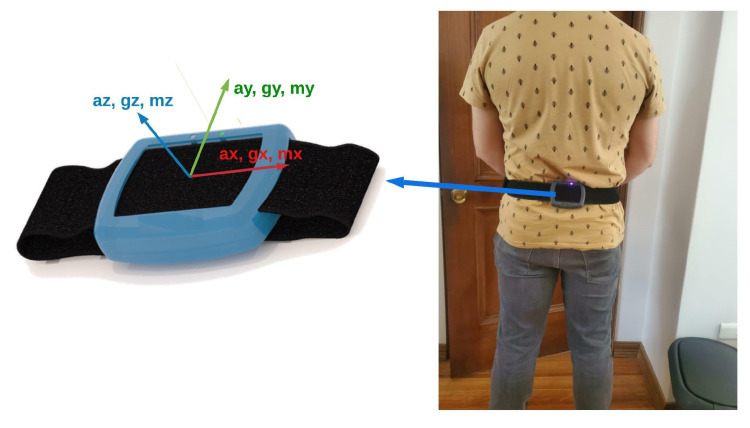
Sensor location for human movement analysis. The sensor is located to deliver their positive measurements in movements according to the axis.

To acquire significant movement information from the patients using a single measurement unit, we locate the MIMU on the lower back (L3–L5) using a 50 mm wide hook and loop elastic band with an adjustable design (see Figure 3). The final developed sensor has a weight of 104 g and a size of 95 × 75 × 20 mm.

The implemented sensor can measure static and dynamic movement information with low error in applications where the drift and noise produced by this sensor are negligible for human movement analysis purposes (see Table 1).

### 4.2. Mobile App for Motion Analysis

The measurement platform is a mobile application that communicates with the wireless movement sensor using Bluetooth 5.0.

**Figure 4:**
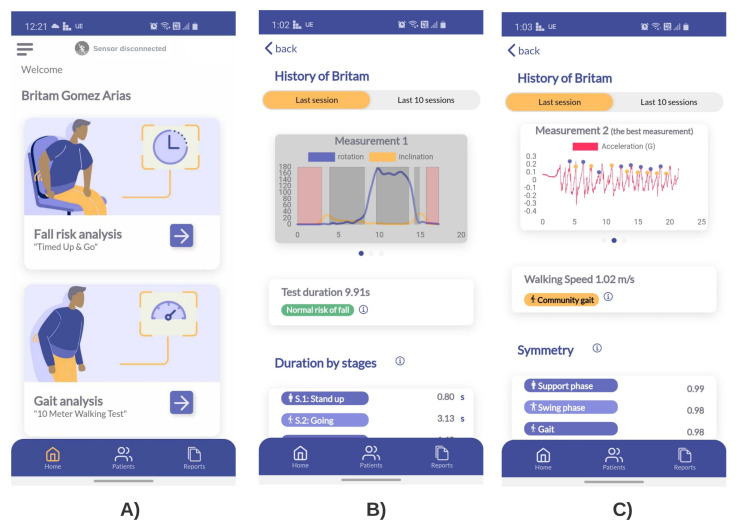
Mobile app implemented for home-care human movement analysis. Note that there are both tests for risk of falls and gait analysis. (**A**) Home menu to select the assessment test. (**B**) iTUG automatic results. (**C**) iT10M automatic results.

The mobile app incorporates two algorithms developed and validated previously by the research team of this work. The first assess the risk of falls through the segmentation of the instrumented Timed Up & Go test (iTUG) [26], and the second is used for the analysis of gait through the instrumented 10 m walking test (iT10M) [27] (see Figure 4).

The mobile app communicates with an application programming interface (API) to manage the database where the user’s pre-selected data are stored. We implemented an API using the PHP framework Lumen Laravel version 5.8. This API contains all the information related to the user sessions, the list of subjects, the raw signals (accelerations, angular velocities, and orientations), and the movement parameters extracted from each instrumented assessment scale.

#### 4.2.1. Instrumentation of the Timed up & Go Test

**Figure 5:**
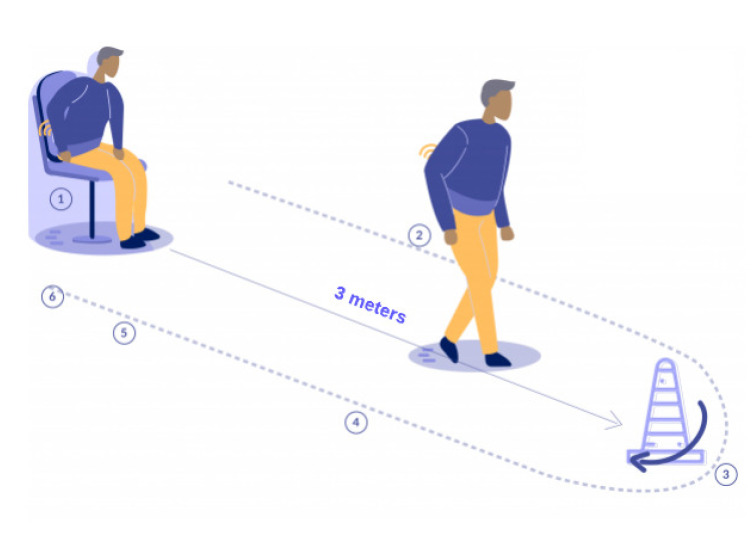
iTUG test with each phase segmented: (1) Standing, (2) Go walking, (3) 3 m mark turning, (4) Return walking, (5) Turn before sitting, and (6) Sitting. Note that the sensor is located on the lower-back of the person.

Although the iTUG is relatively new. Different authors indicate that the phases to be measured in the iTUG test are: (a) Standing, (b) Go walking, (c) 3 m mark turning, (d) Back walking, (e) Turn before sitting, and (f) Sitting (see Figure 5).

The automatic identification, segmentation, and analysis of the phases of the iTUG can increase its predictive value for the risk of falls. This approach produces a segmented evaluation regarding the phases in which the subjects present significant difficulties. We proposed an algorithm to automatically segment each phase of the iTUG using inertial data (accelerations and angular velocities) and orientation data (quaternions) [26]. Those algorithms allow us to extract of temporal indexes such as the duration of each iTUG phase and mobility parameters. The parameters we evaluate are the acceleration profile during standing and sitting, velocity profile during standing, sitting, and turning, steps during walking and trunk inclination during sitting and standing phases. Table 2 summarises the information that can be obtained using the proposed system.

#### 4.2.2. Instrumentation of the 10-m Walking Test

**Figure 6:**
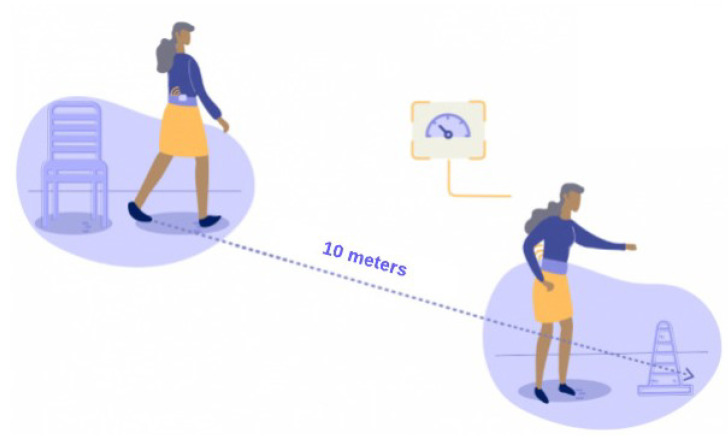
iT10M test. Note the sensor is located on the lower-back of the person.

The iT10M is a clinical test used to evaluate the patient’s gait from the performance of the walk in a predetermined distance of 10 m. In addition, the walking speed is measured and correlated with the patient’s level of independence (see Figure 6). The instrumentation of this test allows estimating gait parameters with a single sensor located on the lower-back of the patient using a wearable alternative.

The algorithm for gait analysis used and validated by the research team [27] allows processing the tri-axial accelerometry signals from the lower back. During the execution of the gait test, these data permit identifying the events of initial contact and final contact of each foot and, in this way, identifying the main gait events. With such data, it is possible to extract the spatio-temporal parameters of the gait cycle from each limb: Cadence (step/min), step time (s), stride time (s), double support time (s), single support time (s), swing phase duration (%), stance duration (%), step length (m), and stride length (m). Table 3 summarises the information that can be obtained using the proposed system.

## 5. Applying DnD to the Proposed Movement Analysis System for Home Care

Based on the aforementioned wearable movement analysis system (cf. Section 4), this research keeps developing and validating our DnD-based methodology and fosters a home-care project on multi-purpose daily activity monitoring and analytics. In particular, three main purposes of this home-care project are (1) establishing new indicators of individual health by monitoring a population’s everyday activities, (2) measuring individual health by monitoring an individual’s one-day activities, and (3) detecting/predicting individual accidents by monitoring an individual’s real-time activities. As a matter of fact, it has been identified that dynamic factors such as physical activities should be considered alongside the static indicators (e.g., body mass index) to better reflect a person’s health status [48].

Benefiting from our developed devices and trial studies, we are able and ready to monitor and identify a wide range of human activities. In particular, the DnD-friendly analytic methods formulated in Equations (4)–(7) have been extensively used in our current monitoring trials (e.g., for real-time statistics of standing, sitting, and walking duration). Although monitoring the skewness of those indicators has not been implemented in our current work, we still report on this analytic method (i.e., Equation (9)) for the purpose of making this paper self-contained. By obtaining these parameters and indexes from a large of healthy people, we will further be able to create standard indicators and in turn use them for individuals’ health measurement and fall risk detection.

### 5.1. DnD-Based Privacy Preserving Monitoring and Analytics of Daily Activities

**Figure 7:**
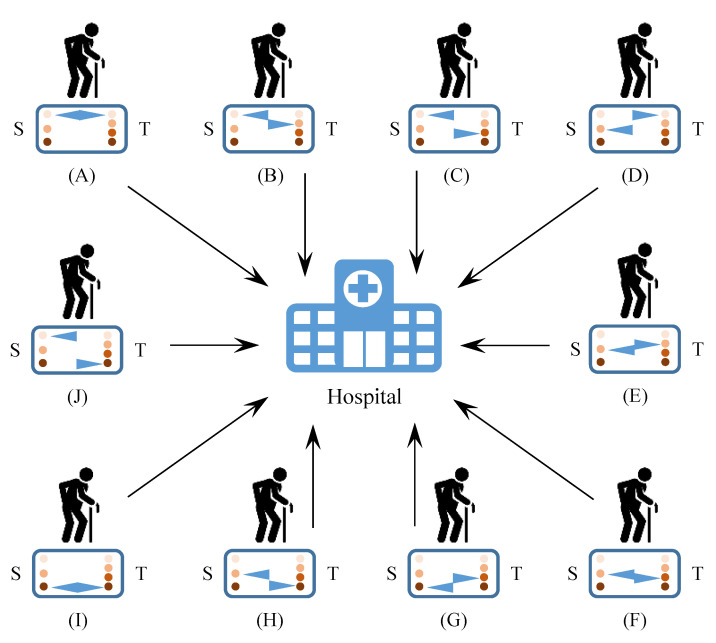
Different daily activity monitoring scenarios in the simplified healthcare network. In particular, *S* indicates user control policies for data storage, while *T* indicates user control policies for data transmission. (A) Store nothing and transmit nothing. (B) Store nothing and transmit regular handshakes and/or occasional notifications. (C) Store nothing and transmit analytic result. (D) Store analytic result and transmit nothing. (E) Store analytic result and transmit regular handshakes and/or occasional notifications. (F) Both store and transmit analytic result. (G) Store raw data and transmit analytic result. (H) Store analytic result and transmit raw data. (I) Both store and transmit raw data. (J) Store nothing and transmit raw data.

The DnD approach advocates intrinsic privacy preservation by controlling data sharing and storing. The current implementation of DnD is to relieve the privacy risks in patient-centric monitoring and analytics of daily activities. For the convenience of discussion, we assume that the remote home-care from a hospital is based on a two-tier healthcare network, as shown in Figure 7.

At the home side of the healthcare network, the patient-centric data can be controllable across three storage levels and four transmission levels, respectively, as distinguished in Table 4. Specifically, it is suggested to retain locally either no data, analytic result, or raw data; and allow users to transfer either no data, regular handshakes, analytic result, or raw data to healthcare professionals. Correspondingly, the case study distinguishes between DnD-applicable scenarios and traditional analytics scenarios, as briefly explained below.

The DnD-applicable scenarios are proposed to be suitable for implementing DnD when we can forget the raw data immediately, such as:

- Scenario (A): Store nothing and transmit nothing. In this scenario, the collected raw data and the analytical result of a user’s daily activities are neither saved by the user nor shared with the others. Technically, we can follow the moving-window and/or the running statistical methods to continuously summarise the user’s daily activities. The summary can be viewed at any time on a user-side monitor, while the present values will automatically vanish (or be updated) when the user refreshes the summary another time. In fact, this has been a common self-monitoring practice at home, e.g., tracking steps and heart rate from a smart watch.
- Scenario (B): Store nothing and transmit regular handshakes and/or occasional notifications. On top of Scenario (A), this scenario allows the hospital (who supervises the home care) to receive some basic information from the user at home. The regular handshakes may indicate the proper running status of the home-based daily activity monitoring system. The occasional notifications can be different shortcut codes that represent various predefined alerts. Once particular alerts are triggered, the user will receive professional interventions from the hospital. For example, if a fall is predicted or detected, the responsible professional will be notified and the remote camera will automatically be connected under the user’s pre-granted permission to further decide whether or not to send an ambulance.
- Scenario (C): Store nothing and transmit analytic result. Depending on the user’s control, the statistical summaries of a user’s daily activities can be transmitted to the hospital either automatically or manually on a daily basis. By treating the hospital as a trusted third party (TTP), the user can let the hospital maintain his/her activity-related health profile instead of saving the daily summaries locally. Note that the mechanism of occasional notifications is enabled by default in this scenario. It is also worth mentioning that the user’s living habit will not be disclosed in those overall summaries, because the detailed time series of the activity indicators and parameters are not recorded at all.
- Scenario (D): Store analytic result and transmit nothing. Similar to Scenario (A), here, the user also self-monitors the daily activities at home. Furthermore, in contrast with Scenario (C), the user is responsible for maintaining the activity-related health profile for himself/herself. Given the full control over the local data, the user can freely remove the historical summaries of daily activities, for example, to save storage space. On the other hand, since the hospital receives nothing, no professional intervention will be involved in this scenario.
- Scenario (E): Store analytic result and transmit regular handshakes and/or occasional notifications. This scenario obtains combinatory benefits from Scenarios (B) and (D). By saving the daily summaries, the user can maintain his/her activity-related health profile locally. By notifying the hospital with predefined alerts, the user will receive remote checkup or on-site care when abnormal activities are predicted or detected.
- Scenario (F): Both store and transmit analytic result. This scenario makes redundant collections of the statistical summary of a user’s daily activities. As a result, the in-home system and the hospital both keep a copy of the user’s activity-related health profile. From the user’s perspective, this can be for the purpose of backing up or self-tracing the historical records of the summarized daily activities.

In contrast, the traditional analytic scenarios indicate the situations in which we need to store and/or transmit original sensor signals, such as:

- Scenario (G): Store raw data and transmit analytic result. By continuously recording and accumulating the original sensor signals with timestamps, this scenario may enable daily activity mining for a user via his/her in-home system. However, similar to Scenario (C), the statistical summaries of daily activities will be sent to the hospital only without being saved locally.
- Scenario (H): Store analytic result and transmit raw data. On the contrary of Scenario (G), here, the original sensor signals are directly relayed by the in-home system to the hospital, while the statistical summaries of daily activities are saved by the user only. In this case, by centralising different people’s raw data together, the hospital as a TTP will be able to mine regular patterns of daily activities for a particular population.
- Scenario (I): Both store and transmit raw data. This scenario makes redundant collections of the original sensor signals of a user’s daily activities. This may be useful for the user to conduct short-term daily activity analytics and for the hospital to conduct long-term and population daily activity analytics.
- Scenario (J): Store nothing and transmit raw data. By doing nothing locally but relaying the original sensor signals of a user’s daily activities to the hospital, this scenario matches the situations of real-time and remote monitoring in the context of healthcare provisioning [49].

**Figure 8:**
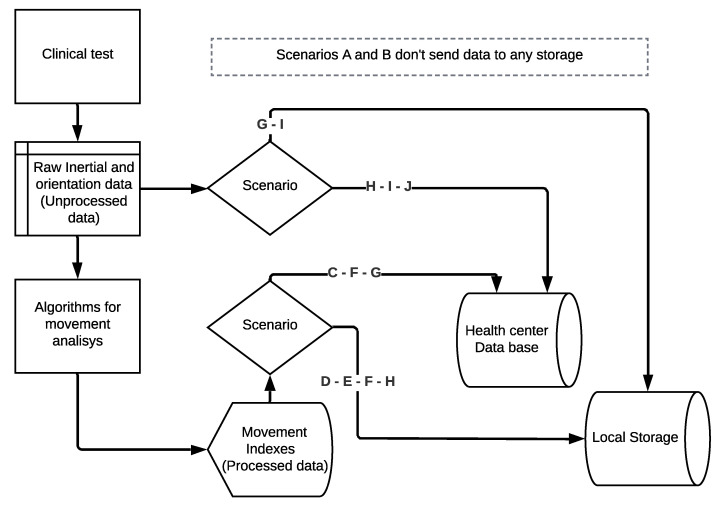
Information flow management depending the scenario selected on the mobile application.

As the Figure 8 scheme shows, the mobile application summarises all the scenarios and controls the information flow.

### 5.2. Technical Implementation and Discussion

To accelerate the project progress, this research has implemented the DnD mechanism into a hybrid microservice system by reusing the software modules from the device-oriented trial studies and by using our previous microservice templates [50] for new functionality development. The whole solution logic is described in Algorithm 1.

The wearable devices send sensor signals to the software system of the local broker via Bluetooth 5.0 up to a maximum distance of 20 m without risk of occlusion. In terms of the mobile application, the user can select in a configuration screen his default privacy scenario and, also, can select his preference each time a clinical test is performed. In practice, the broker can further pass on the raw data to a user-side computer to facilitate local analytics. Given the structured switch-case decision tree, as shown in in Algorithm 1, it is convenient to distinguish between different scenarios and to conduct corresponding analytics. For example, we keep counting the elapsed time a patient spends in sitting, follow Equation (6) to calculate the running average of the patient’s inclination angle when standing up, and follow Equation (7) to calculate the running standard deviation of the patient’s walking speed, without necessarily waiting until finishing a whole-day measurement. Note that the measurable parameters and indices are specified in Section 4.
**Algorithm 1** Solution to daily activity analytics     **Input:** Continuous signal data *S* from the wearable devices.     **Output:** Daily activity analytic results *AR*.1:▹ Firstly, according to the user-controlled policy, try DnD-applicable analysis and also accumulate the raw data.2:D←∅                                                                                              ▹*D* represents the raw data set.3:**for each**s∈S**do**4:    ▹ Decompose each signal *s* into a tuple of meaningful information pieces $\left(d_{1},d_{2},d_{3}\ldots \right)$.5:    $(d_{1},d_{2},d_{3}\ldots )\leftarrow s$6:    **if** DnD-applicable **then**7:        ▹ Applying DnD analytic methods (cf. Section 3.1) and continuously updating the analytic results *AR*.8:        AR←DnD($d_{1},d_{2},d_{3}\ldots$)9:    **end if**10:    ▹ Optional raw data accumulation (also depending on the predefined policy).11:    $D\leftarrow D\cup (d_{1},d_{2},d_{3}\ldots )$12:**end for**13:▹ Secondly, if the user-controlled policy does not indicate any DnD-applicable scenario, then the analytic results *AR* will be empty, which means the offline analytics will be expected based on the accumulated raw data *D*.14:**if***AR* is NULL **then**                                                                          ▹ Not in DnD-applicable scenarios.15:    AR←OfflineAnalytics(*D*)16:**end if**17:▹ Thirdly, the post-analysis activities will be triggered according to the predefined policy.18:**switch***policy***do**                                                                                 ▹ Given predefined policies.19:    **case** *Store nothing* and *transmit analytic result*20:        D←∅                                                                                      ▹ Erase raw data locally.21:        send(*AR*)22:    **case** *Store analytic result* and *transmit nothing*23:        D←∅                                                                                      ▹ Erase raw data locally.24:        save(*AR*)25:    **case** *Store raw data* and *transmit analytic result*26:        save(*D*)27:        send(*AR*)28:    **case** *Store nothing* and *transmit raw data*29:        send(*D*)30:        D←∅                                                                                      ▹ Erase raw data locally.31:    **case** *Others*32:        ▹ More cases can be involved according to different policies.33:**return***AR*

### 5.3. Privacy Quantification in Different Scenarios of Daily Activity Analytics

Although the aforementioned scenarios of daily activity analytics can be informatively explained via natural-language descriptions, it is not straightforward for users to easily understand and compare the privacy levels between different scenarios. To facilitate privacy recognition and comparison, the developed risk-driven privacy quantification framework (cf. Section 2.2) was applied to this case study, which in turn validates the framework’s efficacy in practice. In particular, the framework distinguishes between users’ privacy sensitivity about data storage and about data transmission. Given the truth that the stored data are still at the user side, the sensitivity scale is assumed to be s=8 for data storage privacy. In contrast, since users will lose control of the data after transmission, the highest sensitivity scale (i.e., s=10) is set for data transmission privacy. For the similar reason, we set the inflection points to be p=50 and p=30 for more-controllable data storage and less-controllable data transmission, respectively, and using the equation system (9) to quantify the privacy in DnD-based daily activity analytics. It should be noted that these parameter settings can conveniently be adjusted on a case-by-case basis as long as their justifications are consistent in/for the same data analytic project (e.g., daily activity analytics in this work).

(9) $$\left\{\begin{array}{c} P_{storage}=1-\mathrm{sig}\left(x;8,50\right)\\ P_{transmission}=1-\mathrm{sig}\left(x;10,30\right)\\ P_{DnD}=P_{storage}\times P_{transmission} \end{array}\right.$$

When it comes to the degree of data disclosure, further assumptions for the two cases of Regular Handshakes and Analytic Result are made, respectively. In the former case, although the regular handshake signals do not include any activity data, they may have carried some basic and predefined user information, e.g., the patient’s identification at least. Therefore, it is aggressively assumed that 10% of personal data can be exposed by the handshake signals. In the latter case, although analytic results have abstracted original data into high-level summaries and interpretations, it is still possible to reveal some raw data details. In fact, recovering information from data summaries has been formally defined as an inverse problem [51]. Moreover, there is no doubt that the more analytic methods are employed together, the more original information could be revealed from the analytic results. For the purpose of demonstration, we fix the data disclosure degree in our case study’s analytic results to be 30% of the entire raw data. Then, the privacy in different scenarios of daily activity analytics can be quantified as shown in Table 4.

It should be noted that the single-dimensional privacy can directly be recognised as 1 if disclosing zero percent of data (i.e., storing/transmitting nothing), or as 0 if disclosing 100% of data (i.e., storing/transmitting raw data), without necessarily using Equation (2) to calculate. In addition, Scenario (—) in Table 4 indicates the situations that are only for theoretical discussions here, because it makes little sense to keep all the data while not utilising them at all.

### 5.4. Technological Appreciation Survey

To know the health-domain professionals’ perception of our technological proposal and methodology for privacy preservation, an anonymous survey of five questions was answered by 16 health professionals (3 medical technologists, 2 nurses, 1 psychiatrist, and 10 physiotherapists). All the 16 health professionals have experience using the movement analysis sensor and did not participate in any instance prior to the development and validation of the proposed technology. In addition, before answering the survey, users had to read and sign the informed consent, which was approved by the Biosecurity, Bioethical and Ethical Committee of the University of Concepción (Number 3180551). The result is presented in Table 5. Regarding whether the proposed technological system for the analysis of human movement is beneficial, 13 health professionals answered “Agree” or “Totally Agree”. At the same time, two indicated “Neither Agree or Disagree”, and one indicated that it did not seem helpful, although the proposed tool is of interest. Regarding whether the privacy preservation proposal is useful for clinical applications, all 16 respondents indicated “Agree” or “Totally Agree”. On whether the proposed method was considered good enough to maintain the privacy of patient information, 15 responded “Agree” or “Totally Agree” while one indicated “Disagree”. About if the proposed methodology has advantages over other existing methods to preserve the information privacy, 12 declared “Agree” or “Totally Agree”, while four answered “Neither Agree or Disagree”. Finally, on whether they would like to see this privacy preservation methodology in other systems or technologies, 15 responded “Agree” or “Totally Agree”, while one indicated “Neither Agree or Disagree”. The results of this survey suggest that the proposed methodology has a high potential from the point of view of professionals in the health area.

## 6. Conclusions and Future Work

This paper presents a novel privacy preservation and quantification methodology for addressing privacy concerns in the home-care environments. In brief, this methodology advocates less sharing and less storing (i.e., DnD) to naturally reduce the significant privacy risks in data transmission and storage. When it comes to less sharing, data distribution has already been identified privacy-friendly for reducing data transmission and increasing user control. As for less storing, we argue to obey the decay theory to “forget” original data after their usage so as to avoid unexpected data leaks even from their owners. In practice, a home-based patient can choose which information he/she wants to share with health professionals according to their mutually agreed private scheme. Moreover, a risk-driven privacy quantification framework can be used not only to help judge the efficacy of DnD-based privacy preservation but also to better satisfy the diverse needs of privacy measurement. As the major contribution of this research, our proposed methodology essentially offers a generic approach to scoping a unified landscape for investigating privacy-friendly home-care systems. By including more data-processing dimensions and by analysing more privacy risks, this methodology will become more and more applicable to guide both the research and engineering of privacy preservation in the home-care environments.

To facilitate validating the proposed methodology, we also developed a movement analysis system for home-based fall risk evaluation and gait monitoring, using a single inertial measurement unit and a mobile analytics app. The custom platform contains algorithms developed and validated for the instrumentation of the iTUG and the iT10M test. The system allows collecting the precise raw data in-home from the wireless movement measurement sensor and processing the collected data to obtain mobility indexes. This in-home evaluation can be done without attending a health centre or using high-technological systems for movement analysis. Thus, we also consider this movement analysis system as our contribution of this research, as it demonstrates the implementation of a lightweight home-care system, in addition to the validation of our methodology.

By introducing our work to several home-care-oriented projects, we have consulted the local professionals and received mostly positive feedback about our privacy preservation and quantification solution. On one hand, this strengthens our confidence that such a distributed and disposable principle will suit more home-care and particularly one-off healthcare scenarios. On the other hand, we realise that our methodology still suffers from some limitations at this current stage. Particularly, the effect of DnD’s privacy preservation largely depends on the application scenarios. For example, we can expect the best privacy-preserving effect only when the raw data are neither stored nor transmitted. Although DnD is compatible with the existing privacy preserving mechanisms, there is still a lack of guidelines for integrating other techniques into DnD to satisfy the needs of mixed home-care scenarios. Therefore, in our future work, we plan firstly to explore new DnD-applicable home-care opportunities and make broader impacts in practice. Secondly, following the fundamental privacy principle (i.e., data minimisation [32]), we plan to combine DnD with the existing privacy preserving mechanisms. By giving DnD the highest priority, DnD will be able to work with and facilitate the implementations of other privacy-preserving techniques.

## Figures and Tables

**Table 1:** Results for the static and dynamic evaluation of the sensor. Static evaluations where performed over 140 min of continuous measurement.

Sensor	Axis/Orientation	RMS	Drift	Offset	Dynamic Error
Acelerometer	X	0.0011 G	-	0.013 G	-
	Y	0.0011 G	-	0.033 G	-
	Z	0.0014 G	-	0.150 G	-
Gyroscope	X	1.12°/s	-	-	-
	Y	1.42${}^{\circ }$/s	-	-	-
	Z	1.41${}^{\circ }$/s	-	-	-
Euler	Yaw	-	0	-	0.25 ± 0.36${}^{\circ }$
	Pitch	-	2.88 $\times \;10^{-6}$${}^{\circ }$/s	-	0.47 ± 0.53${}^{\circ }$
	Roll	-	1.60 $\times \;10^{-5}$${}^{\circ }$/s	-	−0.74 ± 1.92${}^{\circ }$

**Table 2:** Available information in the proposed movement analysis system by the iTUG for risk of falls evaluation.

Personal Data	Raw Data	Movement Indexes
Name Date of birth Address Phone number Gender Height Weight Email Health history	Tri-axial accelerations (m/s${}^{2}$) Tri-axial angular velocities (degrees/s) Normalized quaternion orientations	Risk of falling (Normal, Risk, High Risk) Test duration (s) Standing duration (s) Sitting duration (s) Walking duration (s) Turning duration (s) Pre-sitting turning duration (s) Standing max acceleration (m/s${}^{2}$) Sitting max acceleration (m/s${}^{2}$) Maximum inclination during standing (degrees) Maximum inclination during sitting (degrees) Number of steps during walking Angular velocity during turning (degrees/s) Angular velocity during pre-sitting turning (degrees/s)

**Table 3:** Available information in the proposed movement analysis system by the instrumentation of the iT10M for gait analysis.

Personal Data	Raw Data	Movement Parameters
Name Date of birth Address Phone number Gender Height Weight Email Health history	Tri-axial accelerations (m/s${}^{2}$)	Functional mobility Walking speed (m/s) Cadence (step/min) Step time (s) Stride time (s) Double support time (s) Single support time (s) Swing phase duration (%) Stance duration (%) Step length (m) Stride length (m) Gait symmetry

**Table 4:** Quantified Privacy in Different Scenarios of Daily Activity Analytics.

Privacy Dimension with respect to Data Storage	Privacy Dimension with respect to Data Transmission			
	**Nothing to Transmit** (0% disclosure)	**Regular Handshakes** (10% disclosure)	**Analytic Result** (30% disclosure)	**Raw Data** (100% disclosure)
**Nothing to Store** (0% disclosure)	Scenario (A): 1	Scenario (B): 0.881	Scenario (C): 0.5	Scenario (J): 0
**Analytic Results** (30% disclosure)	Scenario (D): 0.924	Scenario (E): 0.814	Scenario (F): 0.462	Scenario (H): 0
**Raw Data** (100% disclosure)	* Scenario (—): 0	* Scenario (—): 0	Scenario (G): 0	Scenario (I): 0

* Impractical scenarios for theoretical discussions only.

**Table 5:** Technological appreciation survey.

Item	Strongly Disagree	Disagree	Neither Agree Nor Disagree	Agree	Totally Agree
(1) Are you satisfied with the proposed clinical human motion analysis system?	1	–	2	6	7
(2) Do you consider this privacy-preserving methodology helpful for clinical applications?	–	–	–	10	6
(3) Is the proposed privacy preservation methodology good enough to ensure the privacy of patient information?	–	1	–	9	6
(4) To the best of your knowledge, do you think the proposed privacy-preserving methodology has advantages over other known methods?	–	–	4	6	6
(5) Would you like to see the proposed privacy preservation methodology in other technologies or clinical applications?	–	–	1	6	9
